# High-Throughput Sequencing and Degradome Analysis Identify miRNAs and Their Targets Involved in Fruit Senescence of *Fragaria ananassa*

**DOI:** 10.1371/journal.pone.0070959

**Published:** 2013-08-19

**Authors:** Xiangbin Xu, Lili Yin, Qicai Ying, Hongmiao Song, Dawei Xue, Tongfei Lai, Maojun Xu, Bo Shen, Huizhong Wang, Xuequn Shi

**Affiliations:** 1 College of Life and Environmental Sciences, Hangzhou Normal University, Hangzhou, People's Republic of China; 2 College of Environment and Plant Protection, Hainan University, Haikou, People's Republic of China; National Institutes of Health, United States of America

## Abstract

In non-climacteric fruits, the respiratory increase is absent and no phytohormone is appearing to be critical for their ripening process. They must remain on the parent plant to enable full ripening and be picked at or near the fully ripe stage to obtain the best eating quality. However, huge losses often occur for their quick post-harvest senescence. To understanding the complex mechanism of non-climacteric fruits post-harvest senescence, we constructed two small RNA libraries and one degradome from strawberry fruit stored at 20°C for 0 and 24 h. A total of 88 known and 1224 new candidatemiRNAs, and 103 targets cleaved by 19 known miRNAs families and 55 new candidatemiRNAs were obtained. These targets were associated with development, metabolism, defense response, signaling transduction and transcriptional regulation. Among them, 14 targets, including NAC transcription factor, Auxin response factors (ARF) and Myb transcription factors, cleaved by 6 known miRNA families and 6 predicted candidates, were found to be involved in regulating fruit senescence. The present study provided valuable information for understanding the quick senescence of strawberry fruit, and offered a foundation for studying the miRNA-mediated senescence of non-climacteric fruits.

## Introduction

Fruits are essential component of the human diets, especially from fleshy species, which contain high levels of vitamins, antioxidants and dietary fiber. According to the ripening characteristic, fleshy fruits are designated as climacteric and non-climacteric [1]. Climacteric fruits are characterized by a burst of respiration at the onset of ripening along with a large rise in ethylene production, such as the apple, banana, peach and tomato. They can ripen off the parent plant and get soft and sweet after harvest, and can be picked before the fully ripe stage to maintain quality and extend storage life. Besides, their ripening can be initiated by exposure to exogenous ethylene. In non-climacteric fruits, the respiratory increase is absent and no phytohormone is appearing to be critical for the ripening process, and they can not be initiated by exogenous phytohormone. They must remain on the parent plant to enable full ripening and be picked at or near the fully ripe stage to obtain the best eating quality. However, at ambient temperature, some of the non-climacteric fruits post-harvest senescence occurs very quickly, such as strawberry and cherry, and their storage time is only 2–3 d, which often serious affects fruit quality and marketing value, and causes huge losses. Thus, studies of the complex molecular mechanism of non-climacteric fruits post-harvest senescence and technologies for extending their storage life have attracted considerable attentions.

In recent years, small RNAs (sRNAs) are getting more and more attention for their key roles in post-transcriptional or translational gene regulation [2]–[6]. Small interfering RNAs (siRNAs) and microRNAs (miRNAs) are the two major groups of sRNAs. The miRNAs are short (20 to 24 nucleotides in length), single strand and endogenous non-coding sRNAs molecules that negatively regulate gene expressions at post-transcriptional level identified in nearly all eukaryotes [4], [7]. The miRNA genes originate in the nucleus, where they are encoded by independent transcriptional units in intergenic regions and transcribed by RNA polymerase II to form pri-miRNA. In plant, the stem loop region of pri-miRNAs are cut by Dicer endonuclease to form small double stranded RNA (dsRNA) miRNA: miRNA* and then transported to cytoplasm by HST [4], [8]. Then the miRNA* strand is degraded by SDN and the miRNA strand is incorporated in the RNA-induced silencing complex (RISC) with endonuclease AGO [9], where they serving as a leading RNA to direct cleavage of complementary mRNAs [9]. Recent studies have revealed the key roles of miRNAs in diverse biological processes such as development, hormone response and stresses response [10]–[13]. Several miRNAs also have been found to be effective in regulating different mechanisms entailing plant senescence. For example, miR319 negatively regulates leaf growth and positively regulates leaf senescence by modulating the activity of TCP transcription factors [14]. MiR164 prevents premature overexpression of *ORE1*, a positive regulator of ageing-induced cell death and senescence, and regulates the senescence and cell death in *Arabidopsis thaliana* leaves [15]. In the climacteric fruit of tomato, miR156/157 was found to be complementary to the CNR3′ untranslated-region sequence, which was important for MADS-RIN to induce the transcription of ripening-related genes [16]. However, to date, there is no information about the function of miRNAs in non-climacteric fruits post-harvest senescence.

To totally make clear the functions of miRNA on the diverse biological processes, it is essential to exactly identify their target genes and explore their interactions. Recently, the development of degradome sequencing has provided a new method for validation of the splicing targets on a whole genome scale, which has employed in identifying miRNA targets and revolutionized the traditional computational target prediction. It has been successfully applied to screen for miRNA targets in several plants, animal and viruses [17]–[21]. In the present research, to investigate the molecular mechanism of non-climacteric fruits post-harvest senescence, and screen for miRNAs and their targets involved in senescence, two small RNA libraries and one degradome from different storage time strawberry fruit were constructed and sequenced by the high-throughput Illumina Solexa system. A selected number of strawberry fruit miRNAs were then validated by quantitative RT-PCR. Based on these identified miRNAs, we predicted their potential targets by degradome sequencing and analyzed the senescence mechanism for the first time in strawberry fruit.

## Results

### Analysis of small RNA library data sets

**Figure 1:**
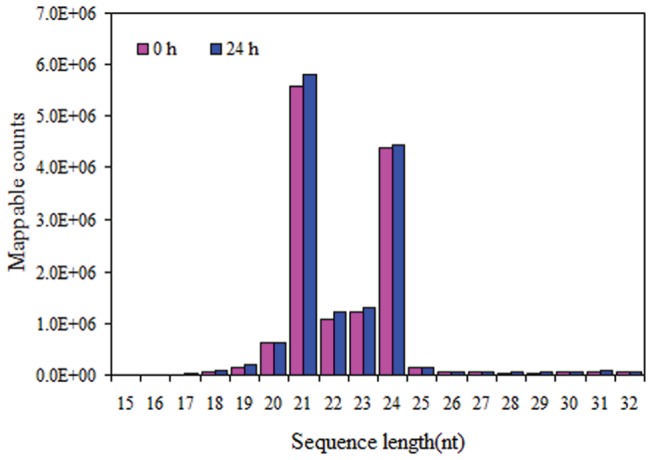
Length distribution of mappable counts of sequ-seqs type in two libraries of strawberry fruit.

For identification of miRNAs and other endogenous small RNAs involved in senescence of non-respiration climacteric fruit, two independent small RNA libraries from strawberry fruits stored for 0 and 24 h were generated and sequenced by high-throughput Illumina Solexa system. These two small RNA libraries yielded a total of 19,440,558 and 20,983,425 raw reads, respectively (Table 1). After filtering out the adapter sequences as well as sequences with low quality or low-copy, and further removing mRNA, rRNAs, tRNAs, snRNAs, and snoRNAs, a total of 18,759,735 and 20,293,492 mappable small RNA sequences were obtained, respectively (Table 1). The cloning frequency of different sized small RNAs (15–32 nt) was similar between the two libraries. In both libraries, the majority of the sRNAs were 20–24 nt in size, two major peaks at 21 nt and 24 nt were observed (Fig. 1).

**Table 1:** Distribution of the small RNA sequences in the two libraries.

	0 h stored fruit		24 h stored fruit	
RNA class	counts	percentage of total	counts	percentage of total
Raw reads	19440588	100%	20983425	100%
Number of reads removed due to 3ADT^a^ not found	33994	0.17%	38677	0.18%
Number of reads removed due to <15 bases after 3ADT cut	57985	0.30%	59400	0.28%
Junk reads	56288	0.29%	58202	0.28%
Number of mappable reads	18759735	96.5%	20293492	96.71%
mRNA^b^	2172202	11.17%	2385965	11.37%
Rfam^c^	3959416	20.37%	4499476	21.44%
Repeats^d^	12538	0.06%	18274	0.09%
Rfam types				
rRNA(45.7%)	1277713	6.57%	1261461	6.01%
tRNA(25.4%)	822581	4.23%	1032868	4.92%
snoRNA(6.2%)	10673	0.05%	13831	0.07%
snRNA(2.4%)	25180	0.13%	34069	0.16%
others(20.2%)	3033413	15.6%	3682276	17.55%

a, 3ADT is the 3′ adaptor.

b: The *Fragaria ananassa* mRNA were downloaded from http://www.rosaceae.org/projects/strawberry genome/v1.0/genes/fvesca v1.0 genemark hybrid.fna.gz.

c: Rfam(V 10.0) were downloaded from ftp://ftp.sanger.ac.uk/pub/databases/Rfam/9.1/.

d: Repeat-repbase (V13.12)were downloaded from http://www.girinst.org/repbase/update/index.html.

### Identification of known miRNAs in strawberry fruit

To identify miRNAs involved in senescence in strawberry fruit, all the mappable sRNAs were mapped to the known plant miRNAs in miRbase (19.0) database. A total of 88 known unique mature miRNAs corresponding to 71 pre-miRNAs with high sequence similarity to the known plant miRNAs were identified (Table S1 and Table S2). Most of these known mature miRNAs were 21 nt in length with the remainder being 20 nt or 22 nt long (Table S1 and Table S2), which is similar to the characteristic of miRNAs from other plant species, suggesting that most of the strawberry fruit known miRNAs are processed by DCL 1 [22]. In these identified known miRNAs, there are 72 known mature miRNAs with 60 novel pre-miRNAs were discovered, among them we found 18 pairs of 5p and 3p miRNAs. These pre-miRNAs cannot be mapped to *Fragaria vesca* genome, but the mature miRNAs can be mapped to the *Fragaria vesca* genome and the extended sequences at the mapped positions of the genome can potentially form hairpins (Table S1). Moreover, 16 mature miRNAs corresponding to 11 reported pre-miRNAs originating from other plant were identified, which can be mapped to *Fragaria vesca* genome. Among them, eight 5p or 3p new mature miRNAs corresponding to reported pre-miRNA in miRbase (19.0) were first time detected in this experiment (Table S2). Base on the sequence similarity, these identified 88 known miRNAs could be grouped into 31 miRNA families. Most of these identified miRNA families such as miR156, miR160, miR164 miR166, miR167, miR168, miR169, miR172, miR390, miR395 and miR399 are highly conserved in a variety of plant species (Table S1). In addition, several known but non-conserved miRNA (miR397, miR535, miR828, miR477, miR479, miR3267 and miR7125) that have previously been identified only from a few plant species were also found in our dataset.

### Identification of novel miRNAs in strawberry fruit

Confident annotation of novel species-specific miRNAs requires *dcl1* or *dcl4* knockout mutants [23], [24]. In the absence of these mutants, the ability of the miRNA flanking sequences to fold-back into a stable hairpin structure is an important criterion for the annotation of new miRNAs [25]. By mapping all unique sRNA sequences to the strawberry genome and predicting the secondary structures of the candidate miRNA precursors, 1157 pre-miRNAs corresponding to 1224 unique mature new candidate miRNAs were first reported in this experiment, of which most were 24 nt in length. These 24 nt length miRNAs were likely to be dependent on DCL3 and the hierarchical action of other DCLs according to the evolution of miRNAs [26]. Among them we found 177 pairs of 5p and 3p new candidate miRNAs. Most of these newly identified miRNAs contained 21–24 nt, and 623 miRNA began with a 5′ uridine (Table S3). The minimum free energy (MFE) of these predicted pre-miRNAs ranged from −14.7 kcal mol^−1^ to −212.9 kcal mol^−1^ with an average of −77.23 kcal mol^−1^, which was similar to the −76.8 kcal mol^−1^ in *Arabidopsis thaliana*, −72.4 kcal mol^−1^ in wheat and −71.0 kcal mol^−1^ in rice, and was much lower than that of tRNA and rRNA [27]. The minimal free energy index (MFEI) ranged from 0.5 to 2.2 with an average of 1.14 (Table S3), which is obviously higher than other types of RNAs such as tRNAs (0.64), rRNAs (0.59) and mRNAs (0.62–0.66) [28]. These characteristic meet the requirements to maintain the stability of hairpin structure of miRNAs.

### Expression profiling of miRNAs in response to senescence

**Figure 2:**
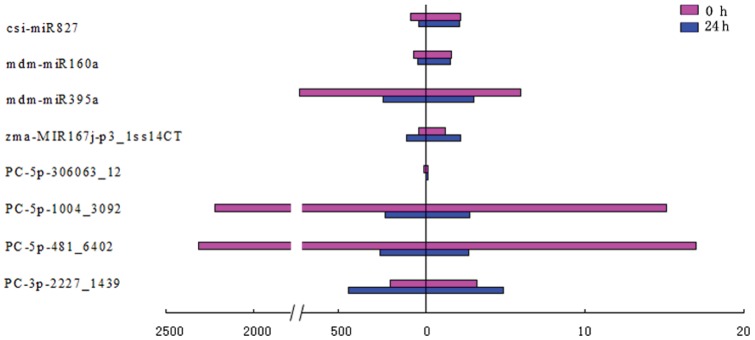
Expression analysis of miRNAs in strawberry fruit stored at 20°C for 0 and 24 h by qRT-PCR. The amount of expression was normalized by the level of *actin 7* in qRT-PCR. All reactions of qRT-PCR were repeated three times for each sample. Left indicates the miRNA relative expression generated from the high-throughput sequencing; Right indicates the miRNA relative expression tested by qRT-PCR.

To identify miRNAs involved in senescence in strawberry fruit, the differential expression of miRNAs in the two libraries was analyzed and compared using the counts of reads generated from the high-throughput sequencing. Considering the extremely low abundances might lead to false results, the known and new candidate miRNAs with less than 10 raw reads in the two libraries were removed from the expression analysis. The expression of miRNAs with log_2_ fold changes higher than 1 was designated as up-regulated, and less than -1 was designated as down-regulated. As showed in Table S5, 158 miRNAs were differentially expressed between the two libraries. Among them, 94 miRNAs were up-regulated and 64 were down-regulated. PC-3p-778131_5, PC-3p-1510675_3, PC-5p-280652_13 and PC-5p-833862_5 had the highest down-regulated fold change. PC-3p-217649_17 had the highest up-regulated fold change in the two libraries. Moreover, some miRNAs both have higher expression and fold changes, such as PC-5p-410_7468, PC-5p-481_6402, PC-5p-1004_3092, gso-miR2109_R+1_1ss14GA, PC-5p-931_3343, mdm-miR395a and PC-3p-8342_429mdm-miR394a were up-regulated, and PC-3p-2227_1439 and gma-miR396a-3p_L+1 were down-regulated (Table 2). There were also most miRNAs with high expression but low log_2_ fold changes, such as mdm-miR156a_L+1, mdm-miR166e, mdm-miR156b, PC-3p-4_731579, ptc-miR156j_1ss15AT, mdm-miR168b, mdm-miR164e, etc. And among them, mdm-miR156a_L+1 had the highest reads abundant (Table 2). To validate the sequencing results and the expression level of miRNAs that involved in fruit senescence, four new candidate miRNAs and five known miRNAs displaying differential expression pattern in strawberry fruits stored for 0 and 24 h from the high-throughput sequencing were selected for qRT-PCR analysis. Although some of them were identified in low read number by Solexa sequencing, such as PC-5p-306063_12 and zma-MIR167j-p3_1ss14CT, all of them were detected by qRT-PCR and they were quite consistent with the results from sequencing data. Moreover, the high abundance miRNAs, such as PC-3p-2227_1439, PC-5p-481_6402, PC-5p-1004_3092, mdm-miR359a, mdm-miR160a and csi-miR827, were also quite consistent with the results from sequencing data (Fig. 2).

**Table 2:** Differentially-expressed miRNAs in strawberry fruit stored at 20°C for 0 and 24 h. (More abundant were shown in Table S9).

miR_name	miR_seq	raw_0 h	raw_24 h	norm_0 h	norm_24 h	log2(24 h/0 h)
PC-5p-410_7468	AGGCTTTGTAGAGGATGGAAT	1626	5149	1490	4357	1.548023
PC-5p-481_6402	TATGTCGCAGGAGAGATGGTAC	257	2389	235	2021	3.104337
PC-5p-1004_3092	TGTCGCAGTAGAGATGGCACG	225	2296	206	1943	3.23757
gso-miR2109_R+1_1ss14GA	TGCGAGTGTCTTCACCTCTGAA	804	1779	737	1505	1.030027
PC-5p-931_3343	TTACCCTTGCAATATCCGTTG	648	1507	594	1275	1.101962
mdm-miR395a	CTGAAGTGTTTGGGGGAACTC	268	859	245	727	1.569174
PC-3p-8342_429	CAGGGCGAATACACTCTCAGA	170	434	156	367	1.234234
PC-3p-2227_1439	TGCTCACTCTTCTTCTGTCAGC	432	214	396	181	−1.12951
gma-miR396a-3p_L+1	GTTCAATAAAGCTGTGGGAAG	247	129	226	109	−1.05199
mdm-miR156a_L+1	TTGACAGAAGAGAGTGAGCAC	1066002	947170	977085	801495	−0.28579
mdm-miR166e	TCGGACCAGGCTTCATTCCCC	404322	542884	370597	459388	0.309862
mdm-miR156b	TGACAGAAGAGAGTGAGCAC	578015	440209	529802	372505	−0.50819
PC-3p-4_731579	TTTGAAGTGGGATTTGGCGAA	237203	332497	217417	281359	0.371947
ptc-miR156j_1ss15AT	TTGACAGAAGATAGTGAGCAC	268627	200977	246220	170067	−0.53384
mdm-miR168b	TCGCTTGGTGCAGGTCGGGAA	36235	58778	33213	49738	0.5826
mdm-miR164e	TGGAGAAGCAGGGCACGTGCA	58872	52595	53961	44505	−0.27795
mdm-miR167g_1ss21AG	TGAAGCTGCCAGCATGATCTG	25764	37618	23615	31832	0.430774
mdm-miR167b_R+1_1ss21AC	TGAAGCTGCCAGCATGATCTCA	37021	31362	33933	26538	−0.35463
peu-MIR2911-p3_1ss4AG	GGCGAGAGCGGGTCGTCGCGT	18189	31186	16672	26390	0.662564
mdm-miR535d_1ss7CT	TGACGATGAGAGAGAGCACGC	30627	27761	28072	23491	−0.25702
PC-3p-132_23619	TCTATTCAAAGAGATGACTGTT	14006	24715	12838	20914	0.704049
PC-3p-28_119226	TGAATTGGGATTTGGCGAATT	20969	20070	19220	16983	−0.17852
PC-3p-391_7887	CGGGCTTGGCAGAATCAGCGGGGA	10231	13828	9377	11701	0.319433
PC-3p-82_35024	CAGGTTGTGAAGGTACTAGCAT	9563	12665	8765	10717	0.290075
han-MIR2911-p5_1ss20TA	CGAACCCGTCGGCTGTCGGAG	4479	8674	4105	7340	0.838398
mdm-miR408b_L-1R+1_1ss8AC	CAGGGACGAGGTAGAGCATGG	4255	5727	3900	4846	0.31332
PC-5p-224_13566	TATTGGCAGACTTGAAGCATT	5091	4598	4666	3891	−0.26205
mdm-miR172g_R+1	AGAATCTTGATGATGCTGCAT	4059	3965	3720	3355	−0.14899
PC-5p-437_6958	AGTGGTATCAGGGCTATGTTA	3997	3570	3664	3021	−0.27839
mdm-miR166a_R+1	TCGGACCAGGCTTCATTCCCCC	1605	2507	1471	2121	0.527947
PC-5p-1433_2156	TTACCTGTAGAAGCGGGAGTGC	1463	2318	1341	1961	0.54828
PC-5p-395_7852	TGTAGAGCCAATGGCTGATCC	1174	1669	1076	1412	0.392062
mdm-miR166b_1ss1TG	GCGGACCAGGCTTCATTCCCC	991	1607	908	1359	0.581781
PC-5p-687_4397	AGTGGAGTTCTGGGAAAGAAG	1696	1537	1554	1300	−0.25747
ptc-miR168b-3p	CCCGCCTTGCATCAACTGAAT	1051	1517	963	1284	0.415037
PC-3p-1366_2253	ATTGGAAAGGTGAGAAGTTGG	1989	1516	1823	1283	−0.50679
PC-5p-2061_1544	GGGTATAGGTGGGTAGGATAG	1038	1452	951	1229	0.369968
PC-3p-2161_1479	CTCTCTCTCGAAAGGACCTCGG	741	1322	679	1119	0.720727

### Targets identification for miRNAs by degradome analysis

**Figure 3:**
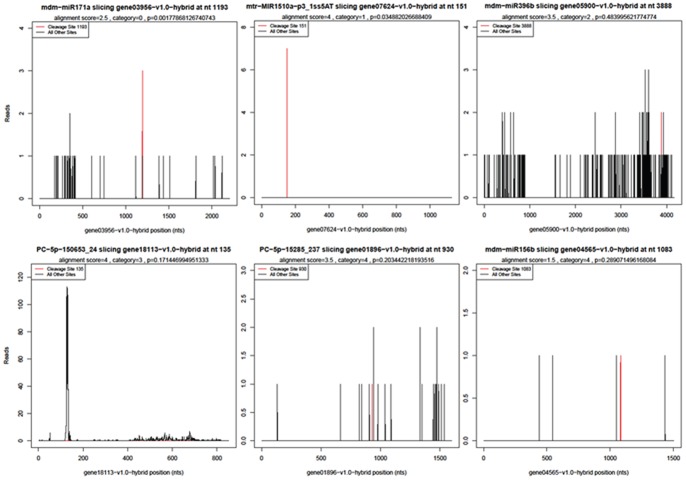
The T-plots of miRNA targets in the five different categories. T-plots show the distribution of the degradome tags along the full-length of the target mRNA sequence. The red line represents the sliced target transcripts. (A) Example of the category 0 target gene03956−v1.0−hybrid for mdm−miR171a. (B) Example of the category 1 target gene07624−v1.0−hybrid for mtr−MIR1510a−p3_1ss5AT. (C) Example of the category 2 target gene05900−v1.0−hybrid for mdm−miR396b. (D) Example of the category 3 target gene18113−v1.0−hybrid for PC−5p−150653_24. (E) Example of the category 4 target gene04565−v1.0−hybrid for mdm−miR156b and gene01896−v1.0−hybrid for PC−5p−15285_237. The categories were based on the relative abundance of the tags at the target sites.

To further understand the biological function of these identified miRNAs on fruit senescence, the high-throughput degradome sequencing approach was adopted to perform a genome-wide analysis of the mRNAs potentially cleaved by the miRNAs. In total, 8,232,621 raw reads from the strawberry fruits libraries (mixture of strawberry fruit stored for 0 and 24 h) were obtained. After removing the reads without the CAGCAG adaptor, 2,921,958 unique raw reads were mapped to the genome database (http://www.rosaceae.org/projects/strawberry_genome/v1.0/genes/fvesca_v1.0_ genemark_hybrid. fna.gz.). The CleaveLand 3.0 was adopted to identify the sliced targets for the known miRNAs and novel miRNA candidates. As shown in Table S6, the cleavage signature for most of the miRNAs were not detectd in this degradome library, only a total of 103 targets potentially cleaved by 19 known miRNAs families and 55 new candidate miRNAs were identified. Based on the ‘height’ of the degradome peak at each occupied transcript position, these cleaved targets were classified into categories 0, 1, 2, 3 and 4 (Fig. 3), respectively. Category ‘0’ is defined as >1 raw read at the position, abundance at position is equal to the maximum on the transcript, and there is only one maximum on the transcript. Category ‘1’ is defined as >1 raw read at the position, abundance at position is equal to the maximum on the transcript, and there is more than one maximum position on the transcript. Category ‘2’ is defined as >1 raw read at the position, abundance at position is less than the maximum but higher than the median for the transcript. Category ‘3’ is defined as >1 raw read at the position, abundance at position is equal to or less than the median for the transcript. Category ‘4’ is defined as only 1 raw read at the position. Among these identified targets, 30 belonged to category 0, one belonged to category 1, 26 belonged to category 2, 1 belonged to category 3, and 49 belonged to category 4, respectively (Table S6).

Based on the BLASTX analysis, 51.5% of the identified miRNA targets detectd in this degradome library were homologous to the genes that have already been found in *A. thaliana* and *Oryza sativa* (Table S6). Some of these genes have been found to be involved in plant senescence, such as auxin response factor, NAC domain transcriptional regulator superfamily protein, growth-regulating factor, alpha/beta-hydrolases superfamily protein, hydroxy methylglutaryl CoA reductase, Myb protein, Pectin lyase-like superfamily protein, Acyl-CoA N-acyltransferases (NAT) superfamily protein, glycosyl hydrolase and beta-galactosidase (Table 3). Moreover, target genes such as trehalose-phosphatase, ARM repeat superfamily protein, disease resistance protein, calmodulin-binding protein and ABA-induced PP2C gene were also identified in the degradome library, which have been reported to be involved in plant stress response.

**Table 3:** Identified miRNA targets involved in fruit senescence by degradome sequencing. (More targets were shown in Table S10).

		Alignment	Alignment	Cleavage			Rep_Norm	
miR_name	Targets	score	range	site	Category	P-value	reads	Annotation
mdm-miR160a,e	gene16844-v1.0-hybrid	1	1956–1976	1967	0	4.62E-03	14	auxin response factor 16
	gene09733-v1.0-hybrid	0.5	1854–1874	1865	0	7.45E-03	28	auxin response factor 16
mdm-miR164d_ 1ss21AC	gene26043-v1.0-hybrid	2.5	650–670	661	0	1.78E-03	42	NAC domain containing protein 38
	gene00971-v1.0-hybrid	3	707–727	718	0	3.20E-03	110	NAC domain transcriptional regulator superfamily protein
	gene04424-v1.0-hybrid	3	653–673	664	0	3.20E-03	4	NAC domain containing protein 87
mdm-miR164e	gene26043-v1.0-hybrid	2.5	650–670	661	0	1.78E-03	42	NAC domain containing protein 38
	gene00971-v1.0-hybrid	3	707–727	718	0	3.20E-03	110	NAC domain transcriptional regulator superfamily protein
	gene04424-v1.0-hybrid	3	653–673	664	0	3.20E-03	4	NAC domain containing protein 87
ptc-miR164f_1ss21TA	gene00971-v1.0-hybrid	4	707–727	718	0	2.49E-03	110	NAC domain transcriptional regulator superfamily protein
	gene04424-v1.0-hybrid	2.5	653–673	664	0	1.78E-03	4	NAC domain containing protein 87
	gene26043-v1.0-hybrid	3.5	650–670	661	0	1.42E-03	42	NAC domain containing protein 38
mdm-miR167b_R+1_ 1ss21AC	gene30394-v1.0-hybrid	4	2606–2628	2618	0	1.25E-03	17	auxin response factor 8
aly-miR169k-3p_L+1	gene19751-v1.0-hybrid	4	366–385	376	4	8.33E-01	1	alpha/beta-Hydrolases superfamily protein
mdm-miR535d_1ss7CT	gene13004-v1.0-hybrid	4	1601–1620	1612	2	6.98E-02	4	hydroxy methylglutaryl CoA reductase 1
hbr-MIR2118-p3_ 1ss1GT	gene12626-v1.0-hybrid	3	1886–1907	1898	4	2.26E-01	1	beta-galactosidase 3
PC-3p-140510_25	gene23708-v1.0-hybrid	4	1394–1414	1405	4	9.49E-01	1	Target of Myb protein 1
PC-3p-29513_122	gene23708-v1.0-hybrid	3	1395–1418	1409	4	7.22E-01	1	Target of Myb protein 1
PC-3p-87823_40	gene00558-v1.0-hybrid	4	936–957	948	4	3.28E-01	1	Pectin lyase-like superfamily protein
PC-5p-1004_3092	gene06191-v1.0-hybrid	4	695–715	706	2	6.98E-02	11	glycosyl hydrolase 9B1
PC-5p-67794_53	gene06057-v1.0-hybrid	3.5	439–459	450	2	1.52E-01	2	alpha/beta-Hydrolases superfamily protein
PC-3p-269545_14	gene11999-v1.0-hybrid	4	415–434	425	2	5.49E-01	3	Acyl-CoA N-acyltransferases (NAT) superfamily protein

### GO function analysis of targets

**Figure 4:**
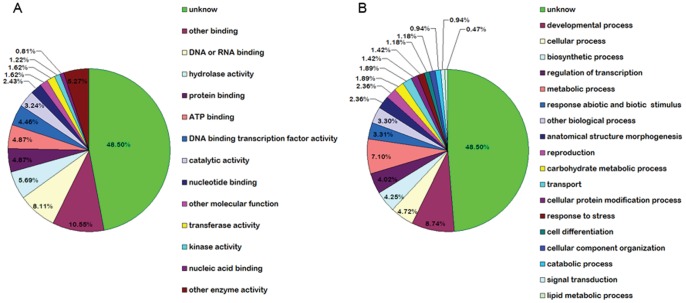
Putative molecular functions (A) and biological processes (B) of known and new candidate miRNA targets. The GO analysis was carried out according to *A. thaliana* and *O. sativa* databases.

To better understand the function of the identified miRNA and reveal the miRNA-gene regulatory network, the target genes obtained from degradome library were subjected to Gene Ontology (GO) analysis based on the *A. thaliana* and *O. sativa* databases. The results of GO analysis showed that the molecular functions of 53 identified targets were involved in DNA or RNA binding, hydrolase activity, protein binding, ATP binding, nucleic acid binding, DNA binding transcription factor activity, catalytic activity, nucleotide binding, transferase activity, kinase activity, other enzyme activity and other molecular function (Fig. 4A). These targets participated in many biological processes, including developmental process, cellular process, biosynthetic process, regulation of transcription, metabolic process, response abiotic and biotic stimulus, anatomical structure morphogenesis, reproduction, carbohydrate metabolic process, transport, cellular protein modification process, response to stress, cell differentiation, cellular component organization, catabolic process, signal transduction, lipid metabolic process and other biological process (Fig. 4B). In addition, the molecular function and biological processes of 50 targets were still unknown.

## Discussion

Strawberry fruit is worldwide appreciated for its unique flavour and high levels of vitamins, antioxidants, dietary fiber, flavonoids, manganese, folate, potassium and ellargic acid (a cancer reduction agent). As the non-climacteric fruit, it must be picked at or near the fully ripe stage to obtain the best eating quality, and huge losses often occur for its rapid post-harvest senescence. Hence, study the mechanisms of strawberry fruit senescence has practical and basic importance. Up to date, many plant miRNAs have been identified and deposited to miRBase, and some of their biological functions also have been studied. To understand the roles of miRNAs in regulation strawberry fruit senescence, two libraries from strawberry fruit stored at 20°C for 0 and 24 h were constructed. Using high-throughput deep sequencing technology, we pyrosequenced sRNA populations at the genome-wide level, and a much wide range of sRNAs with 15–32 nt in length were identified (Fig. 1). According to the analysis from miRBase 19.0, 88 known miRNAs and 1244 new candidate miRNAs were sequenced in the two libraries. By the degradome sequencing approach, we for the first time identified 103 targets, and 53 were homologous to the genes that have already been found in *A. thaliana* and *O. sativa* (Table S6). Most of these homologous genes have already been found to be involved in plant development, stress response, signal transduction, kinase activity and metabolic processes. Fortunately, 17 targets identified in the degradome were found to be related to plant senescence (Table 3).

In *Arabidopsis* leaves, NAC [No apical meristem (NAM), *Arabidopsis* transcription activation factor (ATAF), Cup-shaped cotyledon (CUC)] transcription factor *ORE1* (ORESARA1, oresara means ‘long living’ in Korean), the positive regulator of ageing-induced cell death and senescence, is negative regulated by miR164. During leaf ageing, miR164 progressively decreases and *ORE1* expression increases [15]. In the present study, 3 members of the miR164 family, mdm-miR164d_1ss21AC, mdm-miR164e and ptc-miR164f_1ss21TA were identified in the strawberry fruit (Table S4). Their targets were obtained in the degradome library and annotated as NAC domain transcriptional regulator superfamily protein, NAC domain containing protein 87 and NAC domain containing protein 38 (Table 3). In the process of strawberry fruit senescence from 0 h to 24 h, the reads of mdm-miR164e decreased from 53,961 to 44,505, the reads of mdm-miR164d_1ss21AC decreased from 418 to 406, and the ptc-miR164f_1ss21TA reads increased from 30 to 50 (Table S5). Though there were no significant changes of the log_2_ fold, the significant decrease of mdm-miR164e expression could enhance the expression of NAC domain transcriptional regulator superfamily protein, NAC domain containing protein 87 or NAC domain containing protein 38, and by which they might positively regulate cell death and senescence, and lead to rapid post-harvest senescence of strawberry fruit.

Auxin response factors (ARF) are transcription factors that bind with specificity to TGTCTC auxin response elements (AuxRE) in promoters of auxin response genes and regulate their expression [29], [30]. In *Arabidopsis* leaves, it has been reported that *ARF2*, *7*, and *19* transcripts increased moderately, and *ARF1* transcripts decreased slightly in response to dark induced senescence [31]. *arf2* single mutants also can delay leaf senescence, and in *arf1 arf2* double mutants, this phenotype is enhanced [31]. *ARF6* and *8* have been reported to be targets of miR167, function in promoting both stamen and gynoecium maturation by increasing jasmonic acid (JA) production, and controlling adventitious root initiation by regulating JA homeostasis [29]. *ARF10*, *16*, and *17* are targets of miR160 [32]. In our results, mdm-miR160a,e and mdm-miR167b_R+1_1ss21AC were also identified, and their targets were *ARF 16* and *8*, respectively (Table 3). In the process of fruit senescence, the reads of mdm-miR160a and mdm-miR160e increased from 45 to 70, respectively. In contrast, the reads of mdm-miR167b_R+1_1ss21AC significantly decreased from 33,933 to 26,538 (Table S5). The significant decrease of mdm-miR167b_R+1_1ss21AC expression could increase high expression of *ARF8*, and the increase of mdm-miR160a,e expression could decrease the expression of *ARF 16*. Considering the important roles of *ARF*8 in regulating the production of JA, it must have involved in fruit senescence. The *ARF*16 whether has involved in post-harvest senescence of strawberry fruit, is worthy of our further research.

Myb transcription factors play key roles in regulating diverse transcriptional events in all eukaryotes. *BMyb*, the ubiquitously expressed member of *Myb* gene family, has been reported to be effect in rescuing senescence induced by an activated ras oncogene in rodent cells in vitro [33]. Overexpression of *DMyb* in the imaginal discs of *Drosophila* led to increased levels of apoptosis [34], [35]. Studies on vertebrate Myb family proteins in apoptosis also demonstrated their roles in both pro- and anti-apoptotic [36], [37]. In the present research, the target of Myb protein 1 was identified, which was regulated at different cleavage site by two new candidate miRNA, PC-3p-140510_25 and PC-3p-140510_25, respectively. In the process of fruit senescence, the reads of PC-3p-140510_25 decreased from 47 to 30 (Table S5). We hypothesized that the decrease of PC-3p-140510_25 may play a role in increasing the expression of Myb and promoting senescence in strawberry fruit.

Three hydrolases and 1 lyase were also identified in the degradome library. They were alpha/beta-Hydrolases superfamily protein, beta-galactosidase 3, glycosyl hydrolase 9B1 and Pectin lyase-like superfamily protein, and were regulated by aly-miR169k-3p_L+1, PC-5p-67794_53, hbr-MIR2118-p3_1ss1GT, PC-5p-1004_3092 and PC-3p-87823_40, respectively(Table 3). In the process of strawberry fruit senescence from 0 h to 24 h, the reads of PC-5p-1004_3092 increased from 206 to 1943, and the reads of aly-miR169k-3p_L+1 decreased from 45 to 30 (Table S5). Nevertheless, the reads of PC-5p-67794_53, hbr-MIR2118-p3_1ss1GT and PC-3p-87823_40 showed no significant changes (Table S5). In plants, cell wall plays important role in controlling the cell size and shape. It is also the first obstacle that a pathogen needs to overcome in order to penetrate the plant cell. Its structure is complex and contains various components such as polysaccharides, lignin and proteins. Glycoside hydrolases are common enzymes across all domains of life. They are involved in the metabolism of various carbohydrates containing compounds present in the plant tissues and the degradation and reorganization of cell wall polysaccharides, thereby acting to control fruit softening during ripening [38]. The high level expression of PC-5p-1004_3092 decreased the transcriptions of glycosyl hydrolase 9B1 and might disturb the appropriate balance between degradation and reorganization of cell wall polysaccharides, which probably also related to the quick senescence of strawberry fruit.

Moreover, two senescence relative genes, hydroxy methylglutaryl CoA reductase 1 (HMGR1) and Acyl-CoA N-acyltransferases (NAT) superfamily protein, were identified in the present study, and they were targeted by mdm-miR535d_1ss7CT and PC-3p-269545_14, respectively (Table 3). In the process of strawberry fruit senescence from 0 h to 24 h, the reads of mdm-miR535d_1ss7CT decreased from 28,072 to 23,491, and the reads of PC-3p-269545_14 increased from 10 to 12 (Table S5). In the typical climacteric fruit of tomato, two peaks of *HMGR* mRNA accumulation were observed during early development and ripening, and *HMGR1* and *HMGR2* is responsible for the early development peak correlated with high cell division activity and the ripening-associated peak, respectively [39]–[42]. The melon, another climacteric fruit, which only contains a single *HGMR* gene, also has two-peak expression pattern of *HMGR* gene during fruit development and ripening [42], indicated that *HMGR* was involved in the fruit development, ripening and senescence. In the present study, significant decrease of mdm-miR535d_1ss7CT increased the expression of *HMGR1*, which maybe another mechanism that related to the quick senescence of strawberry fruit.

In summary, for the first time, we obtained a total of 88 known and 1224 new candidate miRNAs from the senescence of strawberry fruit, and identified 103 targets cleaved by 19 known miRNAs families and 55 new candidate miRNAs. Among them, 14 targets cleaved by 6 known miRNA families and 6 new candidate were involved in fruit senescence. These findings provided valuable information for understanding the quick senescence of strawberry fruit and even non-climacteric fruits, and also offered a foundation for future studies of the miRNA-mediated fruit senescence.

## Materials and Methods

### Strawberry fruit material

Strawberry (*Fragaria ananassa* L. cv. Zhangji) fruits were harvested at commercial maturity from an orchard (a private orchard, the owner of Ms. Ma have given permission to conduct the study on this site) in the Xiasha district, Hangzhou, China, and transported to laboratory immediately where they were sorted based on size without physical injuries or infections. The fruits were put in trays with plastic bag to maintain a relative humidity (about 85%), then stored at 20°C for 0, 24, 48 h. No specific permissions were required for these locations/activities, and this study was supported by the National Natural Science Foundation of China (31071836). We confirm that this study did not involve endangered or protected species.

### Total RNA isolation, small RNA library construction and sequencing

The fruits stored at 20°C for 0 and 24 h (the fruit stored for 48 h showed excessive senescence) were collected and frozen in liquid nitrogen immediately, and then stored at −80°C, respectively. Total RNA was extracted using mirVana miRNA Isolation Kit (Ambion, Austin, TX, USA) according to the manufacturer's instructions. Subsequently, equal quantities (10 mg) of small RNA were used for sequencing by the Genome Analyzer GA-I (Illumina, San Diego, USA) following the vendor's instructions.

### Analysis of sequencing data

The small RNA sequences were processed using Illumina's Genome Analyzer Pipeline software to filter out the 5′ and 3′ adapter sequences, and low quantity reads, and then subjected to a series of data filtration steps to obtain mappable sequences using ACGT101-miR v4.2 (LC Sciences, TX, USA). After removed the mRNA and Rfam (rRNA, tRNA, snRNA, snoRNA and repeat sequence), all the trimmed sequences between 15 and 32 bp in length were mapped to miRNA sequences from the miRNA database, miRbase 19.0 (http:// www.mirbase.org/).

To identify potential miRNA precursor sequences, all identified strawberry fruit mature miRNA sequences were BLASTed against the strawberry genome sequences which downloaded from database (http://www.rosaceae.org/). Secondary structure prediction of miRNA precursor was performed by UNAfold software (http://rna.tbi.univie.ac.at/cgi-bin/RNAfold.cgi). In addition, the rest unmapped small RNA sequences were also BLASTed against the strawberry genome sequences and the secondary structure were predicted as above. The non-coding sequences which could form a perfect stem-loop structure and meet the standard of miRNAs prediction [25] were regarded as novel miRNA candidates.

### Verification of strawberry fruit miRNAs by quantitative realtime PCR (qRT-PCR)

To validate the levels of miRNAs, the expression of 8 selected miRNAs were determined by qRT-PCR with SYBR Premix Ex Taq ^TM^ II (TaKaRa, Dalian, China) on the iCycler iQ real-time PCR detection system (Bio-Rad). The specific forward primers of 8 selected miRNAs were designed according to the sequence of miRNA itself (Table S7). Total RNA was extracted from the *F. ananassa* fruit stored at 20°C for 0 and 24 h with TRIZOL reagent following the manufacturer's instructions (Invitrogen). Then the total RNA (1 μg) was treated with DNase I and reverse-transcribed according to the manufacturer's protocol (Transgene, Beijing, China). The relative expression level of miRNA was calculated according to the method of Livak and Schmittgen [43].

### Degradome library construction and target identification

To investigate the potential target mRNAs involved in fruit senescence, a degradome library was constructed from strawberry fruit (mixture of strawberry fruit stored for 0 and 24 h) according to the methods of German et al. [18], [44]. In brief, 1 mg of polyA-enriched RNA was ligated to a 5′ RNA oligonucleotide adaptor which containing a 3′ MmeI recognition site. The first strand cDNA was synthesized by reverse transcription (RT) using the ligated products. Then a short PCR (five cycles) was employed to amplify the cDNA to obtain sufficient quantities of DNA products. After digestion with MmeI and purification with PAGE-gel, the PCR product was ligated to 39-double strand DNA adaptor, and then PAGE-gel purified again for PCR (21 cycles). The final cDNA library was purified and sequenced with a Solaxa/Illumina genome analyzer (LC Sciences, Hangzhou, China). A Public software package, CleaveLand3.0 was used for analyzing sequencing data [17], [45].

### Accession number

Sequencing data obtained in this work have been submitted to the Gene Expression Omnibus under the accession number GSE48055.

## Supporting Information

Table S1 **Profile of known microRNAs originating from pre-miRNAs that cannot be mapped to Fragaria vesca genome,** **but the microRNAs were mapped to the genome and the extended sequences at the mapped positions of the** **genome potentially form hairpins.**
(XLS)

Table S2 **Profile of known microRNAs originating from other plant pre-miRNAs(miRbase 19.0) that can be mapped to** ***Fragaria vesca*** **Genome.**
(XLS)

Table S3 **Profile of new candidate microRNAs originating from predicted RNA hairpins.**
(XLS)

Table S4 **Profiles of all the microRNAs discovered in this experiment.**
(XLS)

Table S5 **Expression profiling of miRNAs in strawberry fruit stored at 20°C for 0 and 24 h.**
(XLS)

Table S6 **Information of miRNA targets by degradome sequencing and GO function analysis.**
(XLS)

Table S7 **Primers used in this study.**
(XLS)

## References

[1] Adams-Phillips L, Barry C, Giovannoni J (2004) Signal transduction systems regulating fruit ripening. Trends Plant Sci 9: 331–338. 15231278 10.1016/j.tplants.2004.05.004

[2] Hamilton AJ, Baulcombe DC (1999) A species of small antisense RNA in posttranscriptional gene silencing in plants. Science 286: 950–952. 10542148 10.1126/science.286.5441.950

[3] Carrington JC, Ambros V (2003) Role of microRNAs in plant and animal development. Science 301: 336–338. 12869753 10.1126/science.1085242

[4] Bartel DP (2004) MicroRNAs: genomics, biogenesis, mechanism, and function. Cell 116: 281–297. 14744438 10.1016/s0092-8674(04)00045-5

[5] Brodersen P, Sakvarelidze-Achard L, Bruun-Rasmussen M, Dunoyer P, Yamamoto YY, et al (2008) Widespread translational inhibition by plant miRNAs and siRNAs. Science 320: 1185–1190. 18483398 10.1126/science.1159151

[6] Lanet E, Delannoy E, Sormani R, Floris M, Brodersen P, et al (2009) Biochemical evidence for translational repression by *Arabidopsis* microRNAs. Plant Cell 21: 1762–1768. 19531599 10.1105/tpc.108.063412 PMC2714937

[7] Llave C, Kasschau KD, Rector MA, Carrington JC (2002) Endogenous and silencing-associated small RNAs in plants. Plant Cell 14: 1605–1619. 12119378 10.1105/tpc.003210 PMC150710

[8] Kurihara Y, Watanabe Y (2004) *Arabidopsis* micro-RNA biogenesis through Dicer-like 1 protein functions. Proc Natl Acad Sci USA 101: 12753–12758. 15314213 10.1073/pnas.0403115101 PMC515125

[9] Baumberger N, Baulcombe DC (2005) *Arabidopsis* ARGONAUTE1 is an RNA slicer that selectively recruits microRNAs and short interfering RNAs. Proc Natl Acad Sci USA 102: 11928–11933. 16081530 10.1073/pnas.0505461102 PMC1182554

[10] Jones-Rhoades MW, Bartel DP (2004) Computational identification of plant microRNAs and their targets, including a stress-induced miRNA. Mol Cell 14(6): 787–799. 15200956 10.1016/j.molcel.2004.05.027

[11] Mallory AC, Vaucheret H (2006) Functions of microRNAs and related small RNAs in plants. Nat Genet 38(Suppl): S31–S36. 16736022 10.1038/ng1791

[12] Sunkar R, Kapoor A, Zhu JK (2006) Posttranscriptional induction of two Cu/Zn superoxide dismutase genes in *Arabidopsis* is mediated by down regulation of miR398 and important for oxidative stress tolerance. Plant Cell 18(8): 2051–2065. 16861386 10.1105/tpc.106.041673 PMC1533975

[13] Voinnet O (2009) Origin, biogenesis, and activity of plant microRNAs. Cell 136(4): 669–687. 19239888 10.1016/j.cell.2009.01.046

[14] Schommer C, Palatnik JF, Aggarwal P, Chetelat A, Cubas P, et al (2008) Control of jasmonate biosynthesis and senescence by miR319 targets. PLoS Biol 6: e230. 18816164 10.1371/journal.pbio.0060230 PMC2553836

[15] Kim JH, Woo HR, Kim J, Lim PO, Lee IC, et al (2009) Trifurcate feed-forward regulation of age-dependent cell death involving miR164 in *Arabidopsis* . Science 323: 1053–1057. 19229035 10.1126/science.1166386

[16] Dalmay T (2010) Short RNAs in tomato. J Integr Plant Biol 52: 388–392. 20377700 10.1111/j.1744-7909.2009.00871.x

[17] Addo-Quaye C, Eshoo TW, Bartel DP, Axtell MJ (2008) Endogenous siRNA and miRNA targets identified by sequencing of the *Arabidopsis* degradome. Curr Biol 18: 758–762. 18472421 10.1016/j.cub.2008.04.042 PMC2583427

[18] German MA, Pillay M, Jeong DH, Hetawal A, Luo SJ, et al (2008) Global identification of microRNA-target RNA pairs by parallel analysis of RNA ends. Nat Biotechnol 26: 941–946. 18542052 10.1038/nbt1417

[19] Ma M, Huang Y, Gong Z, Zhuang L, Li C, et al (2011) Discovery of DNA Viruses in Wild-Caught Mosquitoes Using Small RNA High throughput Sequencing. PLoS ONE 6(9): e24758. 21949749 10.1371/journal.pone.0024758 PMC3176773

[20] Zhao M, Tai H, Sun S, Zhang F, Xu Y, et al (2012) Cloning and Characterization of Maize miRNAs Involved in Responses to Nitrogen Deficiency. PLoS ONE 7(1): e29669. 22235323 10.1371/journal.pone.0029669 PMC3250470

[21] Mao W, Li Z, Xia X, Li Y, Yu J (2012) A Combined Approach of High-Throughput Sequencing and Degradome Analysis Reveals Tissue Specific Expression of MicroRNAs and Their Targets in Cucumber. PLoS ONE 7(3): e33040. 22479356 10.1371/journal.pone.0033040 PMC3316546

[22] Wang L, Wang MB, Tu JX, Helliwell CA, Waterhouse PM, et al (2007) Cloning and characterization of microRNAs from *Brassica napus* . FEBS Lett 581: 3848–3856. 17659282 10.1016/j.febslet.2007.07.010

[23] Ambros V, Bartel B, Bartel DP, Burge CB, Carrington JC, et al (2003) A uniform system for microRNA annotation. RNA 9: 277–279. 12592000 10.1261/rna.2183803 PMC1370393

[24] Rajagopalan R, Vaucheret H, Trejo J, Bartel DP (2006) A diverse and evolutionarily fluid set of microRNAs in *Arabidopsis thaliana* . Gene Dev 20: 3407–3425. 17182867 10.1101/gad.1476406 PMC1698448

[25] Hofacker IL (2003) Vienna RNA secondary structure server. Nucleic Acids Res 31: 3429–3431. 12824340 10.1093/nar/gkg599 PMC169005

[26] Vazquez F, Blevins T, Ailhas J, Boller T, Meins F (2008) Evolution of *Arabidopsis* MIR genes generates novel microRNA classes. Nucleic Acids Res 36(20): 6429–6438. 18842626 10.1093/nar/gkn670 PMC2582634

[27] Yao Y, Guo G, Ni Z, Sunkar R, Du J, et al (2007) Cloning and characterization of microRNAs from wheat (*Triticum aestivem* L.). Genome Biol 8: R96. 17543110 10.1186/gb-2007-8-6-r96 PMC2394755

[28] Zhang BH, Pan XP, Cox SB, Cobb GP, Anderson TA (2006) Evidence that miRNAs are different from other RNAs. Cell Mol Life Sci 63: 246–254. 16395542 10.1007/s00018-005-5467-7 PMC11136112

[29] Guilfoyle TJ, Hagen G (2001) Auxin response factors. J Plant Growth Reg 10: 281–291.

[30] Tiwari SB, Hagen G, Guilfoyle TJ (2003) The roles of auxin response factor domains in auxin-responsive transcription. Plant Cell 15: 533–543. 12566590 10.1105/tpc.008417 PMC141219

[31] Ellis CM, Nagpal P, Young JC, Hagen G, Guilfoyle TJ, et al (2005) AUXIN RESPONSE FACTOR1 and AUXIN RESPONSE FACTOR2 regulate senescence and floral organ abscission in *Arabidopsis thaliana* . Devel 132: 4563–4574. 10.1242/dev.02012 16176952

[32] Rhoades MW, Reinhart BJ, Lim LP, Burge CB, Bartel B, et al (2002) Prediction of plant microRNA targets. Cell 110: 513–520. 12202040 10.1016/s0092-8674(02)00863-2

[33] Masselink H, Vastenhouw N, Bernards R (2001) B-myb rescues ras-induced premature senescence, which requires its transactivation domain. Cancer Lett 171(1): 87–101. 11485831 10.1016/s0304-3835(01)00631-0

[34] Fitzpatrick CA, Sharkov NV, Ramsay G, Katzen AL (2002) Drosophila myb exerts opposing effects on Sphase, promoting proliferation and suppressing endoreduplication. Devel 129: 4497–4507. 10.1242/dev.129.19.4497 12223407

[35] Okada M, Akimaru H, Hou DX, Takahashi T, Ishii S (2002) Myb controls G(2)/M progression by inducing cyclin B expression in the Drosophila eye imaginal disc. EMBO J 21: 675–684. 11847115 10.1093/emboj/21.4.675 PMC125351

[36] Greene LA, Liu DX, Troy CM, Biswas SC (2007) Cell cycle molecules define a pathway required for neuron death in development and disease. Biochim Biophys Acta 1772: 392–401. 17229557 10.1016/j.bbadis.2006.12.003 PMC1885990

[37] Ramsay RG, Gonda TJ (2008) MYB function in normal and cancer cells. Nat Rev Cancer 8: 523–534. 18574464 10.1038/nrc2439

[38] Moctezuma E, Smith DL, Gross KC (2003) Antisense suppression of a β-galactosidas gene (TBG6) in tomato increases fruit cracking. J Exp Bot 54: 2025–2033. 12867545 10.1093/jxb/erg214

[39] Gillaspy G, Ben-David H, Gruissem W (1993) Fruits: a developmental perspective. Plant Cell 5: 1439–1451. 12271039 10.1105/tpc.5.10.1439 PMC160374

[40] Narita JO, Gruissem W (1989) Tomato hydroxymethylglutaryl-CoA reductase is required early in fruit development but not during ripening. Plant Cell 1: 181–190. 2535541 10.1105/tpc.1.2.181 PMC159750

[41] Daraselia ND, Tarchevskaya S, Narita JO (1996) The promoter for tomato 3- hydroxy-3-methylglutaryl coenzyme A reductase gene 2 has unusual regulatory elements that direct high-level expression. Plant Physiol 112: 727–733. 8883384 10.1104/pp.112.2.727 PMC157997

[42] Kato-Emori S, Higashi K, Hosoya K, Kobayashi T, Ezura H (2001) Cloning and characterization of the gene encoding 3-hydroxy-3-methylglutaryl coenzyme A reductase in melon (*Cucumis melo* L. reticulatus). Mol Genet Genomics 265: 135–142. 11370859 10.1007/s004380000401

[43] Livak KJ, Schmittgen TD (2001) Analysis of relative gene expression data using real-time quantitative PCR and the 22DDCT method. Methods 25: 402–408. 11846609 10.1006/meth.2001.1262

[44] German MA, Luo SJ, Schroth G, Meyers BC, Green PJ (2009) Construction of Parallel Analysis of RNA Ends (PARE) libraries for the study of cleaved miRNA targets and the RNA degradome. Nat Protoc 4: 356–362. 19247285 10.1038/nprot.2009.8

[45] Addo-Quaye C, Snyder JA, Park YB, Li YF, Sunkar R, et al (2009) Sliced microRNA targets and precise loop-first processing of MIR319 hairpins revealed by analysis of the physcomitrella patens degradome. RNA 15: 2112–2121. 19850910 10.1261/rna.1774909 PMC2779683

